# Optimization of SA-Gel Hydrogel Printing Parameters for Extrusion-Based 3D Bioprinting

**DOI:** 10.3390/gels11070552

**Published:** 2025-07-17

**Authors:** Weihong Chai, Yalong An, Xingli Wang, Zhe Yang, Qinghua Wei

**Affiliations:** 1 School of Mechanical and Material Engineering, Xi’an University, Xi’an 710065, China; cwh@xawl.edu.cn; 2 School of Mechanical Engineering, Northwestern Polytechnical University, Xi’an 710072, China; anyalong@mail.nwpu.edu.cn (Y.A.); wangxl0525@mail.nwpu.edu.cn (X.W.); yangzhe123@mail.nwpu.edu.cn (Z.Y.)

**Keywords:** hydrogel, extrusion-based 3D bioprinting, parameter optimization, extrusion swelling ratio, fiber formability ratio

## Abstract

Extrusion-based 3D bioprinting is prevalent in tissue engineering, but enhancing precision is critical as demands for functionality and accuracy escalate. Process parameters (nozzle diameter *d*, layer height *h*, printing speed v_1_, extrusion speed v_2_) significantly influence hydrogel deposition and structure formation. This study optimizes these parameters using an orthogonal experimental design and grey relational analysis. Hydrogel filament formability and the die swell ratio served as optimization objectives. A response mathematical model linking parameters to grey relational grade was established via support vector regression (SVR). Particle Swarm Optimization (PSO) then determined the optimal parameter combination: d = 0.6 mm, h = 0.3 mm, v_1_ = 8 mm/s, and v_2_ = 8 mm/s. Comparative experiments showed the optimized parameters predicted by the model with a mean error of 5.15% for printing precision, which outperformed random sets. This data-driven approach reduces uncertainties inherent in conventional simulation methods, enhancing predictive accuracy. The methodology establishes a novel framework for optimizing precision in extrusion-based 3D bioprinting.

## 1. Introduction

Extrusion-based 3D bioprinting technology was first introduced by Landers et al. in 2002 [1]. This technique employs either mechanical or pneumatic systems to extrude bioink through a nozzle or needle, generating continuous microfilaments that are deposited onto a substrate [2]. These filaments rapidly solidify into hydrogel structures, enabling the layer-by-layer fabrication of complex three-dimensional architectures. Compared to inkjet and stereolithography bioprinting methods, extrusion-based printing offers distinct advantages, including a simpler operation, wider material applicability, and higher printing efficiency [3,4,5]. Notably, this technique demonstrates exceptional compatibility with diverse bioink formulations, including cellular aggregates, high-viscosity hydrogels, and acellular matrix components [6]. Currently, this technology has been successfully employed in the fabrication of tissue-engineered constructs, including skin, vascular networks, neural tissues, and cartilage, demonstrating substantial potential for biomedical applications [7,8,9].

In the process of extrusion-based bioprinting, the dimensional accuracy of printed structures represents a core concern for researchers [10,11]. The absence of supporting materials renders printed structures susceptible to gravitational collapse during the printing process, making the design and fabrication of high-precision tissue engineering scaffolds a significant challenge in the field of biomanufacturing [12]. The dimensional accuracy in hydrogel extrusion printing is crucially determined by the physicochemical properties of bioinks. An ideal bioink should exhibit superior extrusion performance, characterized by good fluidity under high shear forces and the rapid recovery of viscoelasticity under low shear forces to maintain structural stability [13,14]. This shear rate-dependent rheological behavior is termed the shear-thinning property [11]. In recent years, researchers have devoted their efforts to developing bioinks with an excellent shape-holding capacity and suitability for bioprinting by exploring diverse matrix materials and their mixing ratios [15,16,17]. Against this backdrop, sodium alginate (SA) has garnered significant attention owing to its exceptional physicochemical and biological properties [18,19]. Sodium alginate, a naturally derived linear polysaccharide extracted from brown algae cell walls, has emerged as a fundamental matrix material in bioprinting applications [20,21]. This biomaterial offers not only outstanding biocompatibility and cost-effectiveness but also demonstrates a rapid crosslinking capability, excellent material compatibility, and tunable rheological and mechanical properties. Nashchekina et al. successfully developed a novel bioink by blending sodium alginate with carboxymethyl cellulose and silk fiberoin, achieving optimal printability, enhanced mechanical properties, and improved structural stability [22]. Scaffolds fabricated with this bioink exhibit superior mechanical characteristics and have demonstrated efficacy in constructing functional tissue-engineered structures. Sofia et al. developed a dual-crosslinked soft gel bioink through the graft modification of sodium alginate, achieving a 5–8-fold increase in storage modulus compared to pure sodium alginate [23]. This bioink can form various geometric structures under a wide range of printing conditions and shows great potential as a template for cell sphere formation.

On the other hand, while the physicochemical properties of bioinks are their inherent attributes, the precise control and optimization of printing processes to enhance printing quality hold broader research value and application potential [24,25]. In the process of 3D bioprinting, the performance of printed structures is significantly influenced by various process parameters, including the nozzle diameter, printing height, extrusion speed, extruder movement speed, printing temperature, scaffold geometry, and porosity [26,27]. In the process of 3D bioprinting, the performance of printed structures is significantly influenced by various process parameters, including the nozzle diameter, printing height, extrusion speed, extruder movement speed, printing temperature, scaffold geometry, and porosity [28,29]. In Stelian et al.’s study, under identical pneumatic pressure conditions, varying the extrusion speeds from 1 mm/s to 20 mm/s resulted in an up to 30-fold difference in hydrogel filament length [30]. Similarly, Tian et al. implemented machine learning to optimize extrusion bioprinting parameters, conducting experiments at 25 kPa and 103.3 kPa pneumatic pressures. The resulting hydrogel fiber diameters differed significantly (752.2 μm vs. 1037.3 μm, a 30% variation), further demonstrating the pronounced impact of process parameters on printing outcomes [31]. Numerical simulation studies have demonstrated remarkable advantages in optimizing printing processes, not only effectively shortening the research and development cycle but also avoiding systematic errors in manual experiments. They provide reliable and theoretical support for the in-depth analysis of bioink flow behavior to improve printing processes [32,33]. For instance, Comminal et al. numerically modeled pressure and velocity distributions within the printing nozzle, establishing the first quantitative correlation between strand spacing, printing/extrusion speeds, and hydrogel deposition quality [34]. Wright et al. implemented computer vision and deep learning algorithms to optimize calibration and printing processes in extrusion-based additive manufacturing [35]. Their dynamic parameter adjustment system achieved high-precision fabrication while enhancing the mechanical properties of printed specimens. Although these studies have achieved preliminary optimization of process parameters, several critical challenges remain unresolved in current research. First, there is still a significant deviation between the simulation model and the actual experiment, making it difficult to accurately simulate the real printing environment [36]. This inevitably leads to errors between the simulation results and experimental results, thus reducing the reliability of the simulation outcomes. Therefore, experimental verification must be conducted to further calibrate the simulation results, which undoubtedly increases the additional workload. Furthermore, most existing studies are confined to the optimization of a single or a few process parameters, while systematic research on the influence of synergistic effects of multiple process parameters on printing quality remains scarce.

This study focused on the optimization of extrusion printing process parameters for sodium alginate–gelatin (SA-Gel) hydrogels. By blending sodium alginate with gelatin to improve the rheological properties of bioinks and based on an extrusion-based printing platform, an orthogonal experimental design was used. A series of printing experiments were systematically carried out to deeply explore the influence of printing process parameters on the forming quality of hydrogels. With the extrusion swelling ratio and fiber formability ratio used as optimization objectives, the optimal combination of these process parameters was successfully carried out, providing an important theoretical basis and practical guidance for the optimization of the extrusion printing process of SA-Gel hydrogel.

## 2. Experimental Setup and Data Validation

### 2.1. Analysis of Hydrogel 3D Printing Process

**Figure 1:**
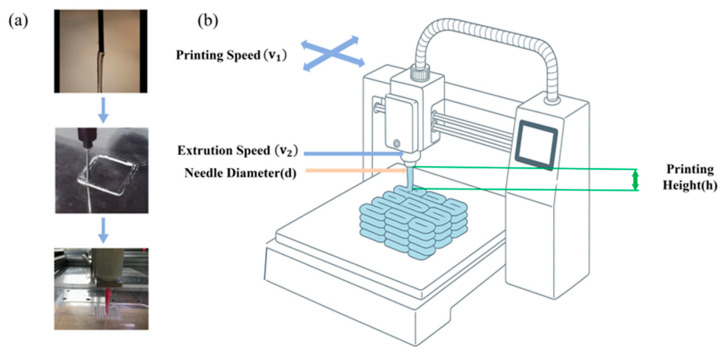
The process of extrusion-based 3D printing. (**a**) The change process of hydrogel fiber. (**b**) Printing process parameters.

During the printing process, the hydrogel is extruded through the nozzle under external force, forming continuous filamentous structures. As fundamental building blocks in 3D bioprinting, the morphological characteristics of fiber filaments have a decisive influence on the forming quality of the final tissue engineering scaffold. Hydrogel with good printability can form fiber filaments with smooth edges and uniform diameter, while hydrogel with poor printability shows rough edges, an uneven diameter, and even presents segmented or droplet shapes [37]. The extruded filaments subsequently deposit onto the printing platform, undergoing a dimensional transformation from one-dimensional strands to two-dimensional structures. However, the deposited hydrogel fibers collapse under mechanical action and gravitational influence, causing the printed lines to deform from circular to elliptical, thereby reducing the height of the layer and leading to printing distortion. Figure 1a illustrates the morphological evolution of hydrogel filaments during extrusion-based 3D bioprinting [38]. The extrusion and deposition characteristics of hydrogels are significantly influenced by varying printing parameters during the bioprinting process [39]. Consequently, four critical process parameters were selected for investigation: the nozzle diameter (d), layer height (h), printing speed (v_1_), and extrusion speed (v_2_), as illustrated in Figure 1b.

### 2.2. Optimal Object

**Figure 2:**
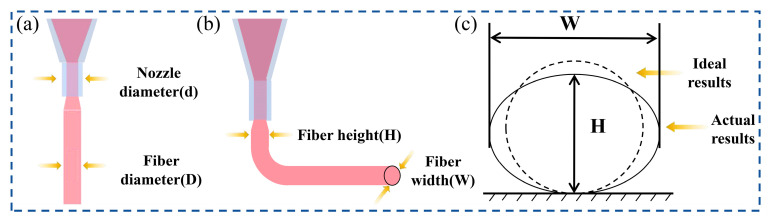
Printing effect evaluation indicators. (**a**) Schematic diagram of extrusion swelling phenomenon. (**b**) Schematic diagram of fiber formability. (**c**) Change in fiber shape after hydrogel deposition.

When hydrogels are extruded through the nozzle under an applied force, they exhibit significant volumetric expansion due to the instantaneous release of shear stress and pressure. This phenomenon is known as the extrusion swelling effect in non-Newtonian fluids (Figure 2a). When the swelling degree is controlled within a reasonable range, the extruded hydrogel filaments can maintain a uniform fiber morphology and exhibit good shape-holding abilities, making them suitable for the printing of subsequent three-dimensional scaffold structures. The extrusion swelling ratio of the hydrogel can be calculated by the following formula:

(1) $$\alpha =\frac{D}{d}$$

where D is the diameter of the fiber filament and d is the diameter of the nozzle. Ideally, the swelling ratio is one, and the closer the extrusion swelling ratio is to one, the better the extrusion effect.

When the hydrogel is deposited on the printing platform, the printed hydrogel filaments collapse downward under the influence of gravity and mechanical forces, as shown in Figure 2b. Increased collapse severity results in greater cross-sectional deviation from ideal circular geometry, directly impairing the dimensional precision of three-dimensional constructs. Consequently, to fabricate high-quality 3D-printed scaffolds, it is essential to minimize line collapse and keep the fiber filaments in an ideal shape close to a cylinder. To quantitatively assess the collapse severity, we defined the fiber formability ratio (β) as the height-to-width ratio of the cross-section:

(2) $$\beta =\frac{H}{W}$$

where W represents the filament width, and H denotes the filament height. Under ideal conditions with perfectly cylindrical filaments exhibiting circular cross-sections, the fiber formability ratio reaches 100%. Actual formability ratios closer to 100% demonstrate reduced filament collapse and superior fabrication outcomes, as depicted in Figure 2c. To comprehensively assess hydrogel performance, two key metrics were established: the extrusion swelling ratio for the extrusion phase and the fiber formability ratio for the deposition stage.

### 2.3. Preparation of SA-Gel Hydrogels

**Figure 3:**
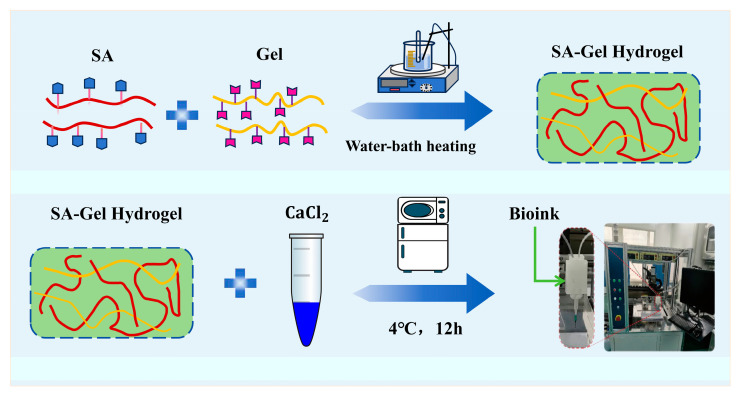
Preparation process of SA-Gel hydrogel.

Figure 3 illustrates the hydrogel preparation process. An appropriate amount of sodium alginate (SA) was dissolved in deionized water at 45 °C and magnetically stirred for 15 min to obtain a transparent solution. Subsequently, a measured quantity of gelatin (Gel) was added, followed by an additional 30 min of stirring to ensure the homogeneous mixing of SA and Gel. Based on prior experimental results, the optimal printability was achieved when the mass fractions of sodium alginate and gelatin in the mixed solution were 6 wt% and 4 wt%, respectively.

### 2.4. Printing Experimental Design and Results

Building upon preliminary simulations and experimental data, this study selected the nozzle diameter d, layer height h, printing speed (v_1_), and extrusion speed (v_2_) for orthogonal experimental design, with each parameter tested at five distinct levels, as specified in Table 1.

**Figure 4:**
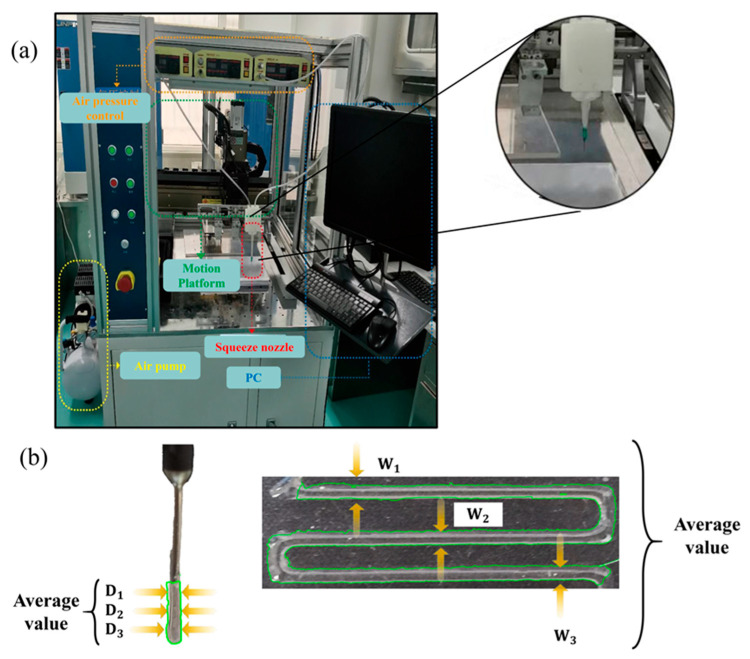
Printing platform and detection method of hydrogel line width. (**a**) Printing platform; (**b**) statistical method for evaluation index data.

Compared with traditional experimental design, the orthogonal test method can significantly reduce the number of experimental combinations and improve experimental efficiency. Therefore, this study adopted the orthogonal test method, designed an orthogonal test table based on the principle of balanced dispersion, and used the level values of each factor as printing experimental parameters to carry out hydrogel extrusion experiments. The printing performance was quantitatively evaluated through the extrusion swelling ratio and fiber formability ratio using a custom-developed extrusion 3D bioprinter, as shown in Figure 4. Prior to printing, bioink was loaded into the pneumatic dispensing system equipped with syringes. The printing model, designed in Solidworks (2020, Dassault Systèmes SolidWorks Corporation, City, France), was converted into G-code via Repetier software (2.3.2, Hot-World GmbH & Co. KG, Willich, Germany) for final printer execution. Following hydrogel extrusion, filament morphology was captured via digital imaging and analyzed using ImageJ software (v1.8.0, National Institutes of Health, NIH, Bethesda, Maryland, USA) to measure the fiber diameter (D). Subsequently, the extrusion swelling ratio was calculated according to Equation (1).

After the hydrogel was deposited, ImageJ software was employed to measure the filament width (W). Since the cross-sectional height of the deposited filaments was difficult to measure directly, the fiber diameter (d) from the extrusion swelling ratio was used as the cross-sectional height (H), and the fiber formability ratio was calculated according to Formula (2). Quantitative results are presented in Table 2 and Table 3, with orthogonal experimental outcomes detailed in Table 4.

The processed results are presented in Table 4. Taking the fiber formability ratio as an example, in Table 1, α_1_~α_5_ represents the fiber formability ratio corresponding to each factor level. The calculation formula is as follows:

(3) $$\alpha_{i}=\bar{\alpha_{ij}}\;i\in (1,5),j\in (1,25)$$

where *j* represents experimental groups with level *i*. The average formability ratio for nozzle diameter level 1 is denoted as an example (0.31 mm diameter).

The range $R_{\alpha }$ of parameter *X* quantifies the formability ratio variation, where greater values indicate a stronger influence. $W_{\alpha }$ is the influence of the weight of each factor on the printing result calculated according to the range, which is the main basis for judging the primary and secondary factors affecting the result. The parameter expressions of $R_{\alpha }$ and *X* are as follows:

(4) $$\begin{array}{l} R_{\alpha }=\max(\alpha_{i})-\min(\alpha_{i})\\ W_{\alpha }=\frac{R_{\alpha ,I}}{\sum_{I}R_{\alpha ,I}}\;I\in [d,h,v_{1},v_{2}] \end{array}$$

According to the data in Table 5 and Table 6, the influential weight of the needle diameter on the extrusion swelling ratio is 43.75%, which dominates the four factors. This indicates that the needle diameter is a key factor affecting the hydrogel swelling phenomenon in the extrusion stage. The influential weight of the extrusion speed was 26.86%, ranking second, while the influential weights of the printing speed and printing height were relatively low, ranking third and fourth, respectively. During deposition, the influential weight of the printing height on the fiber formability ratio was the highest, reaching 34%, followed by the needle inner diameter with a weight of 32.61%. The extrusion speed and printing speed demonstrated lesser impacts at 21.71% and 11.68%, respectively.

### 2.5. Printing Experimental Data Processing

#### 2.5.1. Grey Relational Analysis

Grey relational analysis is a multivariate statistical method that evaluates parameter relationships by assessing the geometric similarity between reference and comparative data sequences [40]. We applied grey relational analysis to evaluate the parameter influences on printing outcomes in this section through the following procedure:

To begin with, the analysis was initiated with data normalization, transforming all values to the [0, 1] range. The extrusion swelling ratio (minimization target) and fiber formability ratio (maximization target) were processed using the following respective formulas [40]:

(5) $$\left\{\begin{array}{c} {x^{*}}_{i}(k)=\frac{\max{x^{o}}_{i}(k)-{x^{o}}_{i}(k)}{\max{x^{o}}_{i}(k)-\min{x^{o}}_{i}(k)}\\ {x^{*}}_{i}(k)=\frac{{x^{o}}_{i}(k)-\min{x^{o}}_{i}(k)}{\max{x^{o}}_{i}(k)-\min{x^{o}}_{i}(k)} \end{array}\right.$$

where ${x^{0}}_{i}(k)$ and ${x^{*}}_{i}(k)$ denote the reference sequence and comparison sequence, respectively. i=1,2,3…,m, and k=1,2,3…,n, where *m* represents the number of target variables and *n* denotes the number of experimental trials.

The grey relational analysis was calculated from normalized data using the following equation:

(6) $$\left\{\begin{array}{l} \gamma (x_{0}^{*}(k),x_{i}^{*}(k))=\frac{\Delta_{min}+\zeta \Delta_{max}}{\Delta_{0i}(k)+\zeta \Delta_{max}}\\ \Delta_{0i}(k)=|x_{0}^{*}(k)-x_{i}^{*}(k)|\\ \Delta_{max}=\underset{i}{\max\;}\underset{k}{\max}\Delta_{0i}(k)\\ \Delta_{min}=\underset{i}{\min\;}\underset{k}{\min}\Delta_{0i}(k) \end{array}\right.$$

In the formula, $\gamma (x_{0}^{*}(k),x_{i}^{*}(k))$ denotes the grey relational coefficient; $x_{0}^{*}(k)$ represents the reference sequence; $x_{i}^{*}(k)$ stands for the comparison sequence; ζ is the distinguishing coefficient (typically taken as 0.5); and $\Delta_{0i}(k)$ is the deviation sequence.

As the evaluation indices for the printing performance included both the extrusion swelling ratio and the fiber formability ratio, it was necessary to calculate the response weights for the two evaluation objectives, with the following calculation method:

(7) $$\begin{array}{l} |\lambda_{k}I_{m}-R|=0\\ a_{k}=\lambda_{k}\left|\sum_{i=1}^{n}\lambda_{i}\right. \end{array}$$

where $I_{m}$ denotes the identity matrix, from which the eigenvalue $\lambda_{k}$ is derived and $\lambda_{1}>\lambda_{1}>\cdot \cdot \cdot >\lambda_{k}>0,k=1,2,3\cdot \cdot \cdot n$ and $a_{k}$ represent the weight values of the objective response.

Finally, the grey correlation degree was calculated through the weighted sum of grey correlation coefficients, with the calculation formula as follows:

(8) $$\gamma (x_{0}^{*},x_{i}^{*})=\sum_{k=1}^{n}a_{k}(x_{o}^{*}(k),x_{i}^{*}(k))$$

#### 2.5.2. Grey Relational Analysis Results

Normalized comparative sequences and reference sequences were substituted into the formula to obtain the deviation sequence. Then, based on the deviation sequence, the average grey correlation coefficients were calculated between the extrusion swelling ratio, fiber formability ratio, and each factor. Principal component analysis and grey correlation degree calculations were performed according to the formula. The final grey correlation analysis results are shown in Table 7.

**Figure 5:**
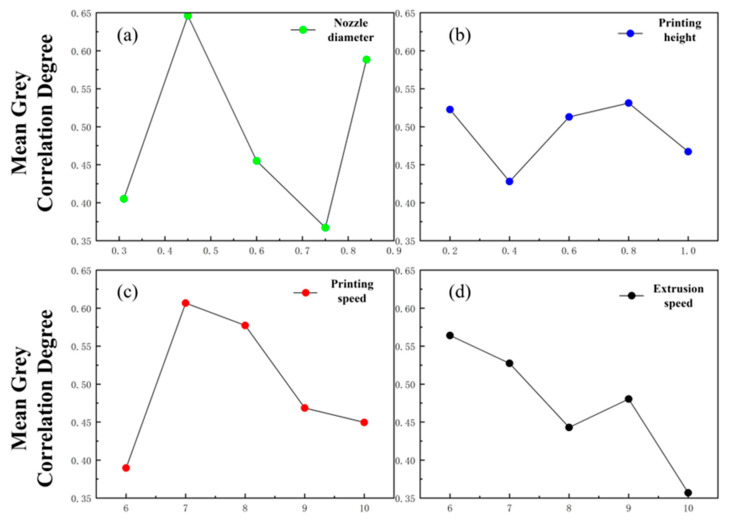
Grey relational main effect diagram of extrusion swelling ratio. (**a**) Grey correlation main effect diagram of needle diameter. (**b**) Print the grey correlation main effect map of the height. (**c**) Grey correlation main effect diagram of printing speed. (**d**) Grey correlation main effect diagram of extrusion speed.

By extracting the grey correlation coefficients of the extrusion swelling ratio and fiber formability ratio for each factor level, the main effect plots of the grey correlation coefficients for the extrusion swelling ratio and fiber formability ratio were obtained, as shown in Figure 5.

Figure 5 shows that the optimal parameter combination for the minimizing extrusion swelling ratio is a 0.45 mm nozzle diameter, 0.8 mm layer height, 7 mm/s printing speed, and 6 mm/s extrusion speed. This configuration achieved peak grey relational coefficients, confirming its optimal performance for target responses. Grey relational range analysis further revealed the nozzle diameter as the most influential parameter for the extrusion swelling ratio, followed by extrusion speed, printing speed, and layer height, which exhibited minimal impact. These findings demonstrate strong concordance with orthogonal experimental results, further validating the reliability of the orthogonal testing methodology.

**Figure 6:**
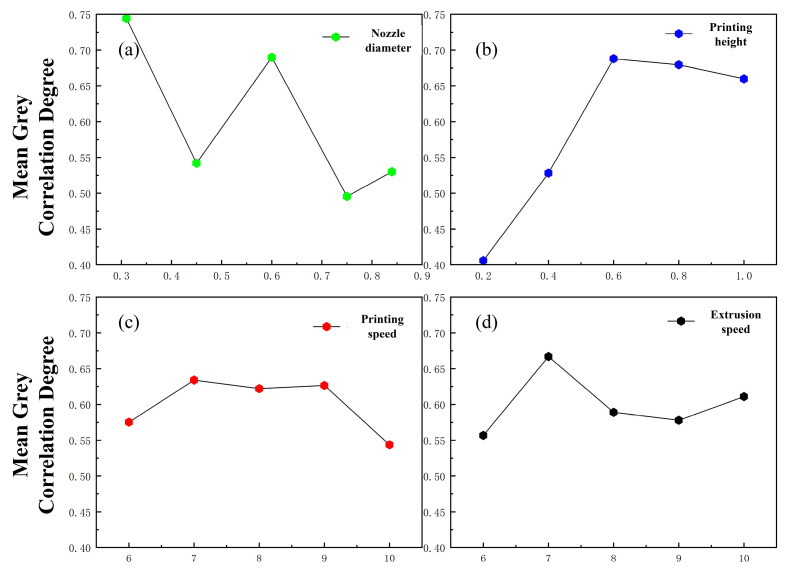
Grey relational main effect diagram of fiber formability ratio. (**a**) Grey correlation main effect diagram of needle diameter. (**b**) Print the grey correlation main effect map of the height. (**c**) Grey correlation main effect diagram of printing speed. (**d**) Grey correlation main effect diagram of extrusion speed.

As indicated by Figure 6, the printing height exerts the most significant influence on the fiber formability ratio, followed by the needle’s inner diameter and then the extrusion speed and printing speed. This finding is consistent with the results of the orthogonal tests described earlier, further validating the correctness of the orthogonal test outcomes. The optimal parameter combination for maximizing the formability ratio is determined as 0.31 mm nozzle diameter, 0.6 mm layer height, 7 mm/s printing speed, and 7 mm/s extrusion speed.

Since the influence of process parameters on the grey correlation degree was a weighted integration of the extrusion swelling ratio and fiber formability ratio, the grey correlation analysis method allowed the composite optimization problem of the extrusion swelling ratio and fiber formability ratio to be transformed into a single optimization problem for the grey correlation degree. By analyzing the variation in the grey correlation degree, the optimization effects of the extrusion swelling ratio and fiber formability ratio under specific printing processes could be intuitively evaluated.

### 2.6. Establishment of Grey Correlation Response Model for Printing Processes

#### 2.6.1. Support Vector Machine Regression Algorithm

**Figure 7:**
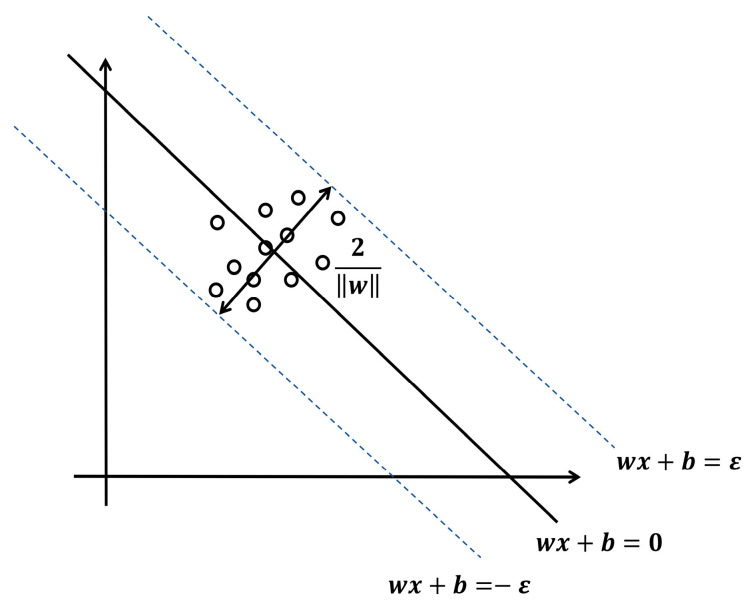
Schematic diagram of SVR model.

The support vector machine algorithm (SVM) was first proposed by Russian scientist Vapnik in 1990 and is mainly used to solve regression or classification problems in practical issues [41]. The support vector regression algorithm (SVR), as the regression variant of SVM, leverages kernel function mapping to effectively handle nonlinear relationships compared with ordinary linear regression, thus avoiding the underfitting problem of traditional linear regression models [42]. In this section, SVR was adopted to establish a prediction model for the influence of printing process parameters on the grey correlation degree, and the schematic diagram is shown in Figure 7.

A training dataset $T=\{(X_{1},Y_{1}),\ldots ,(X_{i},Y_{i}),\ldots ,(X_{n},Y_{n})\}$ was established based on the results of grey relational analysis, where n denotes the size of the training set; $X_{i}=(x_{i,1},\ldots x_{i,j},\ldots ,x_{i,m})$ represents the m-dimensional input sequence; and $Y_{i}$ denotes the actual output value corresponding to this input. The regression function $f(X_{i},w)$ is defined as follows:

(9) $${\hat{Y}}_{i}=f(X_{i},w)=w^{T}\cdot \phi (X_{i})+b$$

where ${\hat{Y}}_{i}$ is the predicted output value; w is the feature weight; b is the bias vector; and ϕ is the nonlinear mapping function.

Subsequently, the penalty factor C and slack variables ${\hat{\xi }}_{i}$ and $\xi_{i}$ were introduced into the regression function to obtain intermediate variables and constraint conditions:

(10) $$\begin{array}{l} \underset{\xi_{i},{\hat{\xi }}_{i},\omega ,b}{\min}\frac{1}{2}||w|{|}^{2}+C\sum_{i=1}^{n}\left(\xi_{i}+{\hat{\xi }}_{i}\right)\\ s.t.\left\{\begin{array}{c} {\hat{Y}}_{i}-Y_{i}\leq \varepsilon +\xi \\ Y_{i}-\hat{Y}\leq_{i}\varepsilon +\hat{\xi } \end{array}\right.,\xi_{i}>0,{\hat{\xi }}_{i}>0 \end{array}$$

where the insensitive coefficient ε is generally set to 0.

Finally, Lagrange multipliers were introduced to solve the feature weights of the regression function, which were then substituted into Equation (9) to establish the regression model as follows:

(11) $$\begin{array}{l} w=\overset{n}{\underset{i=1}{\sum }}(\lambda_{i}-\overset{\wedge }{\lambda_{i}})X_{i}\\ {\hat{Y}}_{i}=\sum_{i=1}^{n}\left(\lambda_{i}-{\hat{\lambda }}_{i}\right)Kernel\left(X_{i},X\right)+b \end{array}$$

where $\lambda_{i}$ and $\overset{\wedge }{\lambda_{i}}$ represent Lagrange multipliers. Meanwhile, to prevent the mapping function from complicating the model solution, the SVR model employed the kernel function $Kernel\left(X_{i},X\right)$ as the nonlinear mapping function for the regression equation.

#### 2.6.2. Development of the Response Model

The SVR model was established on the Matlab 2021b platform, where the input sample data consisted of the normalized process parameters, and the output was the grey correlation degree shown in Table 7. To ensure the reliability of the SVR model, 80% of the 25 sets of sample data were randomly selected as the training set, and the remaining 20% were used as the test set.

**Figure 8:**
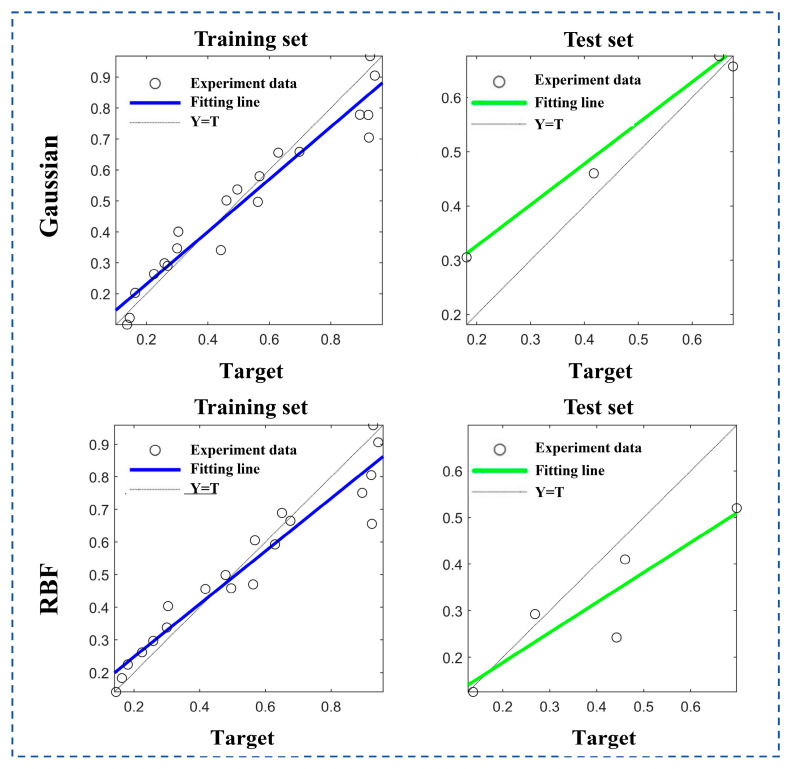
Comparison between Gaussian function model and RBF model.

As established by the grey relational analysis, the relationship between process parameters and the grey correlation degree was nonlinear. Therefore, to obtain a model with better fitting performance, the radial basis function (RBF) kernel and the Gaussian function were separately employed to establish the models. The prediction models established with different functions were evaluated based on their fitting performance and root mean square error (RMSE) for the samples. The results are shown in Figure 8 and Table 8.

**Figure 9:**
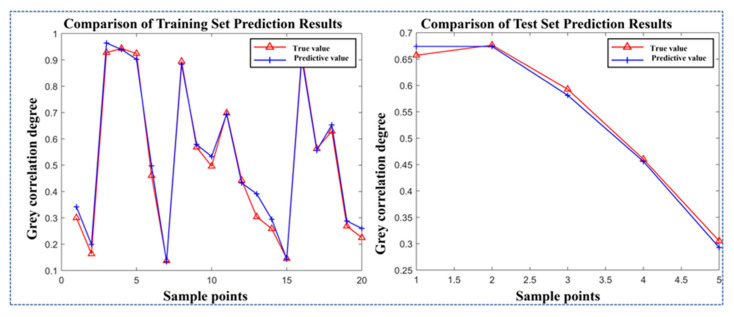
The fitting effect of the SVR model on the training set.

**Figure 10:**
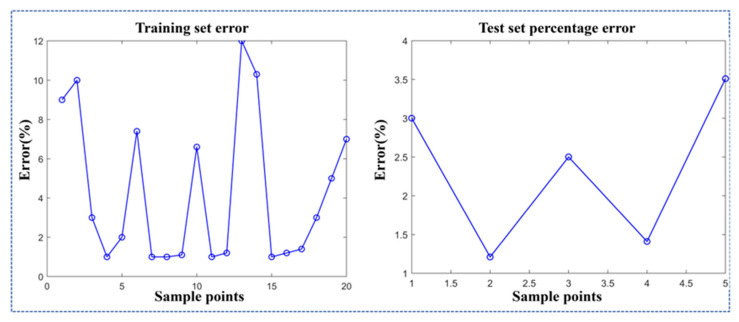
The fitting effect of the SVR model on the test set.

The experimental data demonstrate that the SVR model employing a Gaussian kernel outperforms the RBF-based SVR model across all evaluation metrics, including fitting accuracy, the root mean square error, and the mean absolute error in both the training and testing datasets. Consequently, the Gaussian kernel was selected for the SVR model to perform comprehensive sample analysis and prediction, with the predictive results illustrated in Figure 9 and Figure 10.

As established by Figure 8 and Figure 9, the SVR model utilizing the Gaussian kernel demonstrates prediction accuracy with percentage errors ranging from 1% to 11.8% for both training and testing sets, confirming the reliability of the model. In comparison, Li et al. developed a numerical model integrating printing speed, pneumatic pressure, and layer height to predict dimensional accuracy in extrusion 3D bioprinting; their model exhibited significantly higher prediction errors (5.7–29.8%) and inferior accuracy relative to our proposed mathematical framework [10]. Similarly, Shah et al. employed experimental methods to optimize critical extrusion 3D printing parameters (nozzle diameter, printing speed, layer height, etc.) for precise hydrogel filament dimensional control. However, their model exhibited a filament width prediction error of approximately 15%, exceeding the maximum 11.8% error of our proposed model [43].

### 2.7. Optimization of Optimal Process Parameter Combination

The Particle Swarm Optimization (PSO) algorithm was originally developed by Professor Kennedy and Professor Eberhart in 1995 [44]. The PSO algorithm achieves global optimization through collaborative interactions and competitive mechanisms among individual particles. Unlike alternative optimization algorithms, PSO operates without requiring the gradient computations of the objective function, consequently decreasing iteration counts and enhancing convergence efficiency [45]. The algorithmic implementation proceeds as follows:

In a D-dimensional search space (where D represents the number of parameters to optimize), a swarm comprises N particles, each represented as a D-dimensional vector. The vector components correspond to the particle’s initial position (parameter combination), formally expressed as follows [46]:

(12) $$X_{i}=(x_{i1},x_{i2},\ldots ,x_{iD}),i=1,2,\ldots ,N$$

The velocity of the i particle is defined as follows:

(13) $$V_{i}=(v_{i1},v_{i2},\ldots ,v_{iD}),i=1,2,\ldots ,N$$

Particle velocities are updated according to the following governing equation:

(14) $$v_{id}=w\times v_{id-1}+c_{1}r_{1}(p_{id}-x_{id})+c_{2}r_{2}(p_{gd}-x_{id})$$

where $v_{id}$ represents the velocity of the i particle moving in the d parameter; w denotes the inertia weight; $c_{1}$ is the individual learning factor; $c_{2}$ is the social learning factor; $r_{1}$ and $r_{2}$ are random numbers between [0; 1]; $p_{gd}$ is the known global optimal solution of the population; and $p_{id}$ is the known individual optimal solution.

Through iterative optimization, the swarm converges to the global optimum, where the final particle positions represent the optimal parameter combination. For the established SVR model, PSO was employed to identify the process parameter combination yielding the optimal grey relational degree. This global optimization problem is formally expressed as follows:

(15) $$\begin{array}{l} Find:x=(A,B,C,D)\\ Maximize:GRG=SVR(x)\\ \begin{array}{cc} Subject & to \end{array}:\left\{\begin{array}{c} 0.1<A<1\\ 0.1<B<1\\ 0<C<10\\ 0<D<10 \end{array}\right. \end{array}$$

The optimization was implemented in MATLAB 2021b with the following configuration: The parameter combination that was optimized is denoted as x, which includes the nozzle diameter *A* (0.1–1.0 mm), printing height *B* (0.1–1.0 mm), extrusion speed *C* (0–10 mm/s), and printing speed *D* (0–10 mm/s). The output of the SVR model was defined using the grey relational grade (GRG). The objective function obtained the global maximum GRG value and its corresponding parameter vector *x*. The PSO algorithm parameters were configured with 50 iterations and an inertia weight of 0.8 to balance global and local searches. Both cognitive and social learning factors were set to two, reflecting the equal importance of individual and collective experience. The swarm consisted of 30 particles initialized with a baseline velocity vector of [2 2 2 2] to promote convergence efficiency.

**Figure 11:**
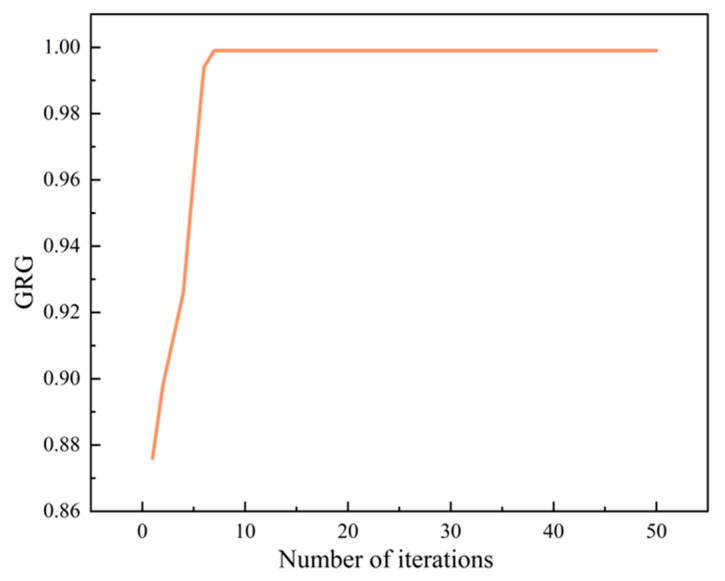
PSO algorithm iteration results.

The iteration results are presented in Figure 11. The PSO algorithm converged to the maximum GRG value by the seventh iteration within the fifty-iteration constraint, demonstrating the appropriateness of the fundamental parameter configuration. The stable convergence indicates the optimal balance between exploration and exploitation capabilities. The optimal GRG of 0.9999 was achieved with the following parameter combination: d = 0.6 mm, h = 0.3 mm, v_1_ = 8 mm/s, and v_2_ = 8 mm/s.

### 2.8. Optimal Printing Process Validation

#### 2.8.1. Printing Preparation

To validate the superiority of the optimized parameters, comparative printing experiments were conducted using both the optimal parameter set and two randomly selected configurations, as detailed in Table 9.

The printing model was designed using SolidWorks software and converted into executable G-code through the Repetier platform (2.3.2, Hot-World GmbH & Co. KG, Willich, Germany). Following printing, the structures were treated with a 2 wt% calcium chloride solution to crosslink the SA, yielding stabilized printed scaffolds with fixed architectures.

#### 2.8.2. Comparison Experiment

**Figure 12:**
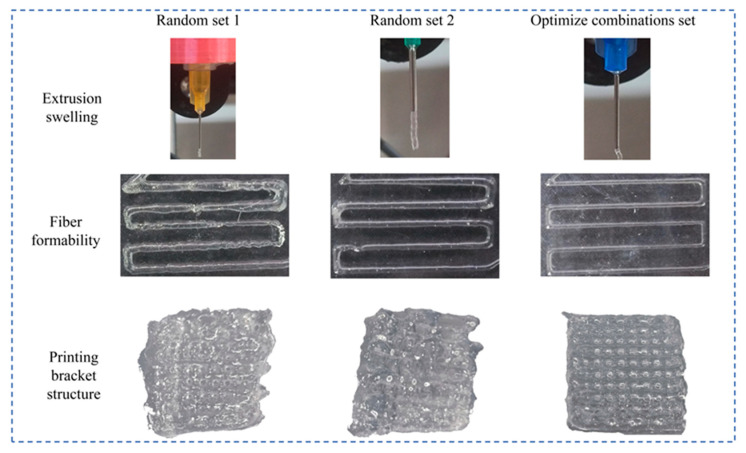
Comparative experimental results.

Printing experiments employing the three parameter sets were evaluated for die swell behavior and filament formation characteristics, with comparative results illustrated in Figure 12. During extrusion, random set one exhibited discontinuous filament formation with segmented structures, while random set two demonstrated an excessive die swell with filament diameters significantly exceeding the nozzle dimensions, both compromising printability. The optimal parameters achieved superior extrusion performance, producing continuous filaments with controlled swelling, which is ideal for precise depositions. During filament deposition, both random parameter sets exhibited filament breakage and printing lag at inflection points, whereas the optimal set demonstrated consistent extrusion without deposition delays. Scaffold fabrication revealed markedly inferior structural integrity with random parameter combinations due to poor filament formability, contrasting sharply with the optimized set’s superior performance. Scaffolds fabricated using random parameter combinations suffered significant structural deformation due to filament merging and collapse, failing to preserve three-dimensional integrity with nearly indiscernible pore architecture. In contrast, scaffolds produced with the optimized parameter combination exhibited excellent structural integrity and well-defined pore architecture. These results conclusively demonstrate the superior printing performance achieved with the optimized parameters, validating the scientific rigor and effectiveness of the preceding parameter optimization methodology.

## 3. Conclusions

Building upon preliminary printing experiments, this study established optimal parameter ranges and implemented an orthogonal experimental design. Subsequently, the multi-objective optimization was transformed into a single-objective problem through grey relational analysis, and a predictive regression model between the grey relational grade and process parameters was established using SVR. The effects of the needle diameter, printing height, extrusion speed, and printing speed on the hydrogel printing performance were systematically investigated. Using the hydrogel extrusion swelling ratio and fiber formability ratio as optimization targets, the optimal process parameters for sodium alginate–gelatin hydrogel extrusion printing were successfully determined. The orthogonal experimental results revealed the following hierarchies for parameter influence. The extrusion swelling ratio is as follows: nozzle diameter > extrusion speed > printing speed > layer height. The fiber formability ratio is as follows: layer height > nozzle diameter > extrusion speed > printing speed. The global optimization of the predictive regression model yielded the optimal parameter combination: 0.6 mm nozzle diameter, 0.3 mm layer height, 8 mm/s extrusion speed, and 8 mm/s printing velocity. Comparative experiments demonstrated the optimized parameter set’s superior printing performance, validating the reliability and accuracy of this data-driven hydrogel parameter optimization approach. This methodology provides scientific guidance for selecting printing parameters in sodium alginate–gelatin hydrogel systems.

## Figures and Tables

**Table 1:** Orthogonal experimental design.

Level Value	Factor			
	d (mm)	h (mm)	$v_{1}$ (mm/s)	$v_{2}$ (mm/s)
1	0.31	0.2	6	6
2	0.45	0.4	7	7
3	0.60	0.6	8	8
4	0.75	0.8	9	9
5	0.84	1	10	10

**Table 2:** Fiber diameter statistics.

d = 0.31	D (mm)	d = 0.45	D (mm)	d = 0.60	D (mm)	d = 0.75	D (mm)	d = 0.84	D (mm)
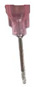	0.343	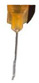	0.510	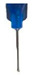	0.670	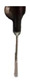	0.817	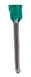	1.025
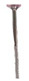	0.332	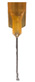	0.540	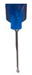	0.650	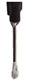	0.767	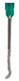	0.867
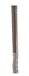	0.322	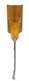	0.549	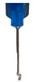	0.660	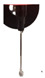	0.800	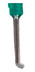	0.894
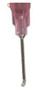	0.34	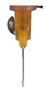	0.468	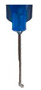	0.610	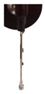	0.767	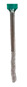	1.000
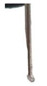	0.325	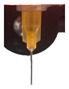	0.462	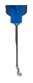	0.690	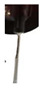	0.850	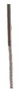	0.921

**Table 3:** Gel filament width statistics.

d = 0.31	W (mm)	d = 0.45	W (mm)	*D* = 0.60	W (mm)	d = 0.75	W (mm)	d = 0.84	W (mm)
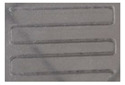	0.551	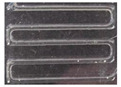	1.043	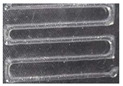	1.595	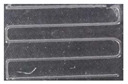	1.246	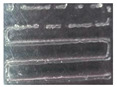	1.569
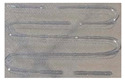	0.385	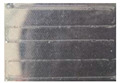	0.717	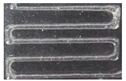	0.940	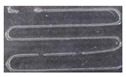	1.361	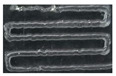	1.562
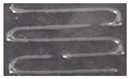	0.739	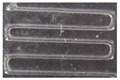	0.780	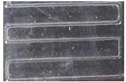	1.113	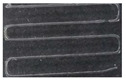	1.125	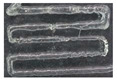	1.509
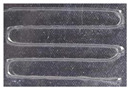	0.409	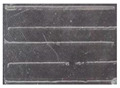	0.612	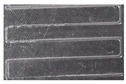	0.734	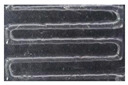	1.475	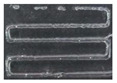	1.634
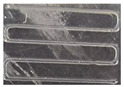	0.444	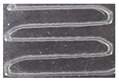	0.853	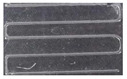	0.883	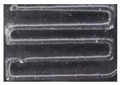	1.486	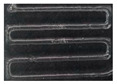	1.426

**Table 4:** Table of orthogonal tests.

Serial Number	Factor				Result	
	d (mm)	H (mm)	$v_{1}$ (mm/s)	$v_{2}$ (mm/s)	α (%)	β (%)
1	1	2	5	3	110.71	62.24
2	1	3	4	5	107.14	86.16
3	1	1	1	1	102.38	43.57
4	1	4	3	2	109.52	83.16
5	1	5	2	4	104.76	73.16
6	2	2	4	2	113.33	48.91
7	2	3	3	4	120.00	62.31
8	2	4	2	1	122.67	70.36
9	2	1	5	5	104	13.69
10	2	5	1	3	102.67	60.36
11	3	1	4	4	111.67	42.36
12	3	4	1	5	108.33	69.16
13	3	2	3	1	110	59.31
14	3	5	5	2	101.67	83.14
15	3	3	2	3	115	78.16
16	4	1	3	3	108.89	23.36
17	4	2	2	5	102.22	31.12
18	4	4	5	4	106.67	53.16
19	4	3	1	2	102.22	59.67
20	4	5	4	1	113.33	63.33
21	5	1	2	2	122.58	35.68
22	5	2	1	4	103.23	59.36
23	5	4	4	3	106.45	59.24
24	5	5	3	5	119.35	66.12
25	5	3	5	1	109.68	40.1

**Table 5:** Table of orthogonal test results (α).

Parameter	Factor			
	d (mm)	h (mm)	v_1_ (mm/s)	v_2_ (mm/s)
$\alpha_{1}$	112.268	109.90	103.77	111.61
$\alpha_{2}$	106.67	107.90	113.46	106.64
$\alpha_{3}$	109.33	110.78	113.55	108.74
$\alpha_{4}$	125.34	110.73	109.74	116.13
$\alpha_{5}$	106.92	108.36	106.55	104.67
$R_{\alpha }$	18.67	2.88	9.66	11.46
$W_{\alpha }$	43.75%	6.75%	22.64%	26.86%

**Table 6:** Table of orthogonal test results (β).

Parameter	Factor			
	d (mm)	h (mm)	v_1_ (mm/s)	v_2_ (mm/s)
$\beta_{1}$	69.658	31.712	54.57	38.16
$\beta_{2}$	51.09	52.188	63.34	55.34
$\beta_{3}$	53.63	65.28	58.85	56.67
$\beta_{4}$	46.12	67.02	60	58.07
$\beta_{5}$	33.68	69.22	50.46	62.112
$R_{\beta }$	35.978	37.51	12.88	23.95
$W_{\beta }$	32.61%	34%	11.68%	21.71%

**Table 7:** Grey relational analysis results.

Serial Number	Result		
	α	β	GRG
1	0.4675	0.60236	0.562589786
2	0.40338	1.00000	0.824056762
3	0.34102	0.45969	0.424694217
4	0.44397	0.92353	0.782107756
5	0.36959	0.93597	0.627924538
6	0.52923	0.49309	0.503747686
7	0.79727	0.60306	0.660332529
8	1	0.69636	0.785903436
9	0.35996	0.33333	0.341183187
10	0.34426	0.98411	0.513378235
11	0.48837	0.45274	0.463247287
12	0.4227	0.68066	0.604587596
13	0.45317	0.57438	0.538635171
14	0.33333	0.92307	0.749155674
15	0.57787	0.81915	0.747996528
16	0.43245	0.36588	0.385511493
17	0.33926	0.39699	0.379965423
18	0.39623	0.52336	0.485869363
19	0.33926	0.57768	0.507369942
20	0.52923	0.61347	0.588627624
21	0.99149	0.41786	0.587023487
22	0.3507	0.57484	0.508741114
23	0.39296	0.57375	0.520435029
24	0.75976	0.64389	0.678060063
25	0.447	0.44031	0.442282881

**Table 8:** Evaluation results of the SVR model.

		Fitting Effect ($R^{2}$)	Rms Error (RMSE)	Average Absolute Error (MSA)
Gaussian function	Training set	0.922	0.061	0.079
	Test set	0.805	0.065	0.079
RBF	Training set	0.907	0.059	0.084
	Test set	0.586	0.926	0.122

**Table 9:** Selection of process parameter combinations.

	d	h	$v_{1}$	$v_{2}$
Random set 1	0.45 mm	0.2 mm	4 mm/s	8 mm/s
Random set 2	0.84 mm	0.4 mm	10 mm/s	5 mm/s
Optimize combinations set	0.6 mm	0.3 mm	8 mm/s	8 mm/s

## Data Availability

The original contributions presented in this study are included in the article. Further inquiries can be directed to the corresponding author.
